# Discrimination and Gender: An Umbrella Review of Psychological Evidence

**DOI:** 10.3390/ijerph23010103

**Published:** 2026-01-12

**Authors:** Giulia Lausi

**Affiliations:** Faculty of Law, Vilnius University, 10221 Vilniaus, Lithuania; giulia.lausi@tf.vu.lt

**Keywords:** social cognition, intersectionality, microaggressions, health inequalities, gender norms

## Abstract

**Highlights:**

**Public health relevance—How does this work relate to a public health issue?**

- Psychological mechanisms driving gender-based discrimination might function as population-level determinants of mental and physical health.
- Collecting systematic evidence on these mechanisms can guide the development of prevention and intervention strategies aligned with public health priorities.

**Public health significance—Why is this work of significance to public health?**

- Discrimination-related mental consequences create substantial, yet preventable, burdens on healthcare systems around the world.
- Addressing the psychological grounds is needed to promote health equity and to mitigate avoidable inequalities.

**Public health implications—What are the key implications or messages for practitioners, policy makers and/or researchers in public health?**

- Policy and practice require integrated approaches aimed to target cognitive, emotional and behavioral dynamics underlying discriminations.
- Advancing research needs rigorous, comparable and intervention-oriented designs, in order to inform effective public health actions.

**Abstract:**

Gender discrimination is a pervasive and multifaceted phenomenon rooted in cognitive, emotional, and social mechanisms that operate across individual, interpersonal, and structural levels. This umbrella review synthesizes systematic reviews and meta-analyses published between 2013 and 2024 examining the relationship between gender, stereotypes, and discrimination. Following PRISMA 2020 guidelines, searches were conducted across PubMed/MEDLINE, Scopus, and Web of Science, yielding 22 eligible reviews after screening 684 records. Thematic synthesis identified two overarching domains: manifestations of discrimination and health and professional outcomes. Discrimination emerged as structural, interpersonal, and implicit, operating through institutional barriers, microaggressions, and stereotyping mechanisms. These dynamics were found to significantly affect mental health, and particularly anxiety, depression, and psychological distress, as well as physical health, including cardiovascular outcomes and maternal morbidity. Professional and social functioning were also impaired, with gender-based inequalities documented in pay, promotion, and role allocation across multiple occupational contexts. Despite consistent evidence of harm, the literature revealed limited consensus in conceptualization and a lack of longitudinal and intervention research. Collectively, findings underscore that gender discrimination constitutes both a public health concern and a systemic social mechanism that shapes individual cognition, emotion, and behaviour, demanding multi-level psychological and policy responses.

## 1. Introduction

Gender discrimination represents a complex and pervasive phenomenon that manifests itself through systematic behaviours, attitudes, and practices favouring or disadvantaging individuals based on their gender identity or gender expression [1]. From a psychological point of view, this phenomenon cannot be understood as an isolated set of discriminatory actions; rather, it needs to be considered within an interconnected network of cognitive, emotional, and social processes that work on individual, interpersonal, and systemic levels [2]. Gender discrimination can be defined as differential treatment, exclusion or disadvantage experienced by an individual based solely on their gender; in psychological research is closely linked to gender stereotypes and bias, which represent underlying cognitive and attitudinal mechanisms that shape expectations and judgments about gendered roles and behaviours [3,4]. These mechanisms often operate automatically and below the level of conscious awareness, translating into discriminatory practices even in the absence of explicit intent [5]. Gender-based discrimination can manifest in both overt forms, such as explicit exclusion or unequal treatment, and subtle ones, including exclusion, biased evaluations or microaggressions [6]. Subtle and automatic forms of discrimination are particularly difficult to detect and address, playing a central role in the persistence of gendered inequalities across social, institutional and occupational contexts [7,8].

Gender, understood not as a biological category but as a multidimensional social and psychological construct, strongly influences the formation of identity, self-perception, and social interactions [9,10,11]. Psychological research has shown how gender discrimination involves basic psychological mechanisms such as social categorization, implicit and explicit stereotype formation, and cognitive bias processes that often operate below the level of awareness. These mechanisms not only perpetuate structural inequalities but also generate significant psychological consequences for the individuals affected by them [12].

The complexity of this phenomenon becomes clear when considering its manifestations in different contexts: from the workplace to the educational environment, from interpersonal relationships to the media, as well as its intersections with other dimensions of identity such as ethnicity, social class, sexual orientation, and age. Many of these psychological mechanisms operate automatically and below the level of conscious awareness. Despite their implicit nature, such processes can nonetheless produce systematic and measurable discriminatory outcomes, shaping evaluations, decisions, and behaviours across individual, interpersonal, and institutional contexts. Gender discrimination does not operate in isolation from society, but is intertwined with other systems of oppression, creating experiences of intersectional discrimination that amplify the negative effects on individual psychological processes [13,14,15]. Moreover, contemporary research has increasingly focused on the temporal dimension of the phenomenon, highlighting how gender discrimination can manifest itself through acute experiences of exclusion or everyday micro-aggressions, generating cumulative effects on psychological well-being over time. This dynamic approach requires a more refined understanding of the mechanisms through which discrimination is transmitted and perpetuated in social contexts [16,17,18].

Given its multiple causes and consequences, the study of the relationship between gender and discrimination in contemporary psychology becomes crucial, developing across different theoretical and methodological levels. From a theoretical perspective, this field of research contributes to advancing our understanding of fundamental psychological processes, from social cognition to identity formation, providing key insights into the mechanisms through which social categories influence individual psychological functioning [19]. Research in this area has fostered the development of new integrative theoretical models that link cognitive, affective, and behavioural processes in the experience of discrimination.

Methodologically, the study of gender discrimination has pushed the psychological discipline towards increasingly sophisticated and multidisciplinary approaches, integrating implicit and explicit measures, qualitative and quantitative methodologies, and longitudinal designs to capture the dynamic complexity of the phenomenon [20]. This has led to the development of more accurate and culturally sensitive measurement tools as well as innovative experimental paradigms to study processes that are typically subtle and automatic.

The social relevance of the issue is even more evident and urgent. Gender discrimination continues to be a major source of inequality in contemporary societies, with direct implications for the mental health, psychological well-being, and quality of life of millions of people [21]. Epidemiological data consistently show higher rates of mood disorders, anxiety disorders, and post-traumatic stress symptoms among groups experiencing gender discrimination, emphasizing the urgency of understanding the underlying mechanisms to develop effective interventions.

From a public policy perspective, psychological research on gender discrimination provides essential evidence for the development of evidence-based prevention and intervention strategies. Understanding implicit bias, for example, has informed the development of bias-reduction training in organizational and educational settings [22]. Similarly, research on resilience and protective factors has contributed to the design of empowerment and psychological support programs.

The social relevance of the topic also extends to its ability to inform public debate and contribute to the development of more equitable and inclusive societies. Psychology, through rigorous research into the mechanisms of discrimination, can provide conceptual and practical tools to counter simplistic or pseudoscientific narratives that often characterize the public debate on these issues. At a time when issues related to gender and identity are at the centre of media and political attention, evidence-based psychological research assumes a crucial role in promoting a more exact and scientifically grounded understanding of these complex phenomena.

## 2. Materials and Methods

This umbrella review was performed in accordance with the Preferred Reporting Items for Systematic Reviews and Meta-Analyses (PRISMA 2020 https://www.prisma-statement.org/prisma-2020-checklist, accessed on 20 May 2025). In accordance with the guidelines and with the FAIR Data Principles and Open Data policy endorsed by European Commission, the protocol and all the related documents have been published on EU Open Research Repository project community (https://zenodo.org/communities/unveilgbd/, accessed on 28 May 2025). Ethical approval is not required for this work, as systematic reviews and meta-analysis only will be analysed. 

### 2.1. Search Strategy

The research has been conducted on PubMed/MEDLINE, Scopus and Web of Science databases, using the search terms: (gender stereotyp* OR gender prejudice) AND (discrimination) AND (systematic review OR meta-analysis OR meta-analysis). The search strategy has been slightly changed according to the different use of Boolean operators within the different databases. The researcher identified systematic reviews and meta-analysis on the relationship between gender stereotypes or prejudices and discrimination. 684 papers were retrieved through search strategy, and 450 records were analysed after duplicates removal.

Literature search has been uploaded on Rayyan to be screened for inclusion and exclusion through a double-blind procedure. Two different reviewers (GL, researcher, and CAB, master’s degree student) used the inclusion criteria to screen the titles and abstracts, to identify all the potentially relevant studies. Full text has been retrieved where the exclusion criteria cannot be determined from the title/abstract screening for final selection. Disagreements were resolved through discussion and consensus.

**Figure 1:**
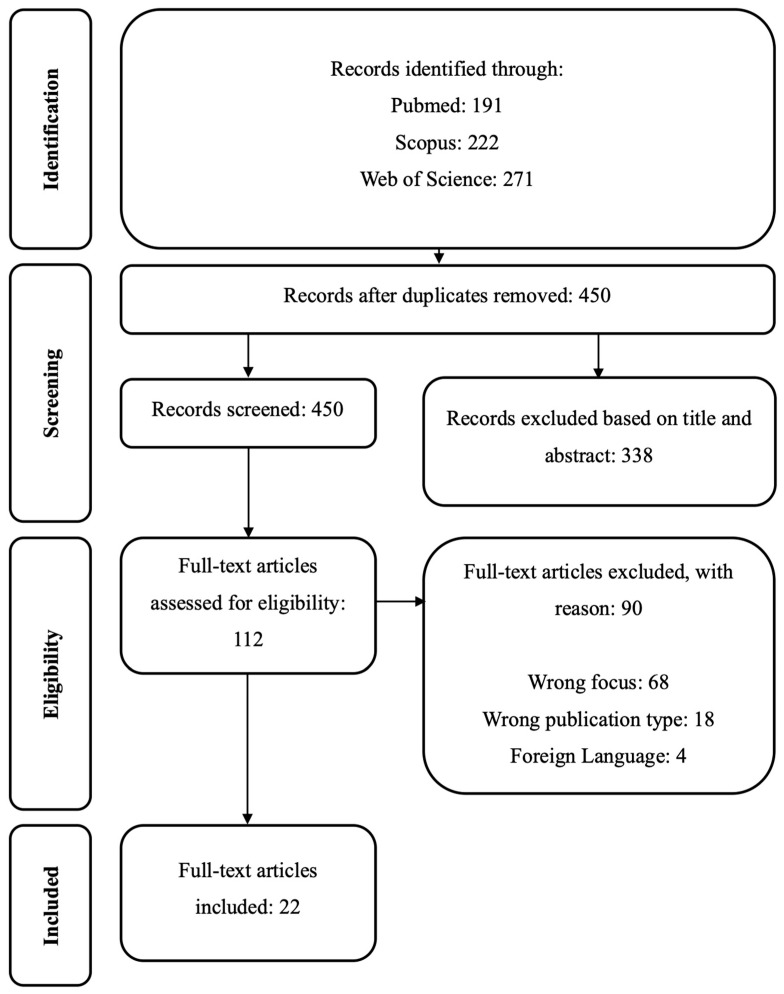
Flow Chart.

Based on title and abstract screening, 338 papers were excluded, while 112 papers were assessed for eligibility. Afterwards, 90 papers were excluded, with reasons (see Flow Chart, Figure 1), resulting in a final number of 22 records reviewed and included in the data analysis.

### 2.2. Inclusion and Exclusion Criteria

Selected articles included English and Italian languages and had to meet the following criteria: (1) being a systematic review or a meta-analysis; (2) article reported data on stereotypes or prejudices directly related to gender-based discrimination. Articles were excluded if they addressed other forms of discrimination (e.g., race, sexual orientation). Papers whose title and abstract mention other forms of discrimination and gender (e.g., sexual orientation and trans people, black people and women) were included only in cases where gender is directly related to discrimination in the study aims.

### 2.3. Risk of Bias Assessment

Risk of bias was evaluated for each systematic review and meta-analysis according to the authors’ own risk of bias declarations for their constituent primary studies. This approach acknowledged the methodological evaluations already undertaken by the systematic review authors whilst recognising the inherent limitations of relying upon secondary assessments. Notwithstanding variations in risk of bias across the included primary studies within each systematic review, all systematic reviews and meta-analyses meeting the inclusion criteria were retained in the final analysis to ensure comprehensive coverage of the available evidence base.

### 2.4. Data Analysis

To extract data from the included paper, researchers developed a data extraction form that included: bibliographical information (author(s), year, title, journal); type of publication (meta-analysis, systematic review); review aim; criteria (inclusion, exclusion); number of included primary studies; synthesis methods and risk of bias assessment; variables; results; limits. The researchers extracted data through double-blind procedure and relied on discussion to resolve disagreements (see Supplementary File S1 for the full final-data extraction table)

To analyse the extracted data, researchers performed a descriptive synthesis to summarise key characteristics of the included reviews (e.g., aims, methodological approaches, number and type of primary studies, outcomes). Findings were grouped thematically according to the research objectives and compared across reviews to identify consistencies, divergences, and research gaps. Quantitative results reported in meta-analyses were summarised narratively, without re-analysis of primary data.

### 2.5. Descriptive Mapping of Selected Papers

The included reviews demonstrated sustained scholarly engagement, spanning publication years 2013–2024. Publications appeared in high-impact, peer-reviewed journals across multiple disciplines, including medical sciences (JAMA Pediatrics, Annals of Surgery), psychological research (Psychological Bulletin), public health (The Lancet Public Health), and social sciences (Health & Place). The methodological distribution comprised systematic reviews (*n* = 13, 59.1%), meta-analyses (*n* = 4, 18.2%), and other review methodologies including scoping, integrative, and narrative approaches (*n* = 5, 22.7%).

Primary study inclusion ranged considerably across reviews (19–129 studies per review), reflecting the extensive empirical foundation underlying gender discrimination research. This variability indicated both the breadth of available evidence and methodological diversity in review scope determination.

Most reviews (*n* = 17, 77.3%) implemented structured quality assessment frameworks, utilizing validated instruments including Joanna Briggs Institute checklists, Newcastle-Ottawa Scale, Cochrane Risk of Bias tool, and AXIS assessment criteria. Consistent limitations emerged regarding moderate-to-low primary study quality, attributed predominantly to cross-sectional designs, self-report measurement dependencies, methodological heterogeneity, and constrained sample sizes.

Research addressed transgender and gender non-conforming youth [23] and adult populations [24,25], sexual minorities [26], and women in son-preference cultural contexts [27]. Investigation extended to corporate leadership discrimination [28], academic research environments [29], competitive eSports [30], and broader societal patterns in Pakistan [31] and China.

### 2.6. Thematic Analysis

Papers have been analysed to identify thematic dimensions within the papers; six dimensions have been identified and, subsequently, collected into two umbrella categories (Table 1). This organisational structure facilitates comprehensive examination of both the methodological rigor and the substantive patterns emerging across diverse research contexts.

## 3. Results

Results of the analysed paper are presented through a systematic synthesis organised along two main thematic dimensions

### 3.1. Theme 1: Gender-Based Discrimination as a Multilevel Process

Theme 1 synthetizes evidence related to gender-discrimination as a multilevel and dynamic process, where cognitive mechanisms, differential treatment and contextual structures interact across individual, interpersonal and institutional levels. These components do not represent isolated phenomena but a sequential and mutually reinforcing pathway through which gender-based inequalities are produced and maintained. The three thematic categories are presented in a sequential order that reflects this process, from the underlying cognitive mechanisms to structural forms of discrimination and to their enactment in everyday interactions.

#### 3.1.1. Implicit Bias and Stereotyping Mechanisms

##### Gender-Related Stereotypes Mechanisms

Gender-related beliefs, stereotypes and implicit biases are shown to be a foundational mechanism underlying discriminatory processes. Gender stereotypes, both descriptive and prescriptive, shape expectations regarding appropriate roles, competencies and behaviours for different gender groups, affecting evaluations and judgements, even without overt discriminatory intent. Descriptive mechanisms refer to beliefs about what people are like according to their gender, while prescriptive mechanisms are related to beliefs on how they should behave, involving pressures to adopt certain behaviours, gender-based role assignments, tokenism, and evaluative judgments [36].

Stereotypes and biases may occur not only explicitly, but also implicitly (e.g., response latencies) and can be influenced by parental attitudes and transmitted to their children, through the observation on nonverbal behaviour or associative learning [37]. According to Degner & Dalege [37], this happens regardless of the gender of either parents or children; however, gender attitudes have been the most extensively studied among the target groups analysed in the meta-analysis. Findings indicate that the effect on gender stereotypes follows the same direction and shows a similar magnitude to that observed for ethnicity, and that these stereotypes are transmitted to a comparable extent in both their affective and behavioural components. This type of evidence is particularly important, as it provides the foundation upon which more “advanced” forms of discrimination are nourished and take root. These dynamics confine individuals to social roles based on their gender. In this cultural imagery, women are framed as an economic burden, whose primary responsibility is caring for the family and the household; men, instead, are constructed as a source of both economic and physical security.

##### Gender Implicit Biases in Everyday Contexts

Implicit biases were described as automatic and often unconscious cognitive processes affecting decision-making, even when there are not explicit prejudices. Such beliefs are not limited to family life but are continuously reinforced by the media, which persist in portraying women in domestic and caregiving roles, as well as by distorted interpretations of religious and cultural teachings [27,31]; evidence from healthcare contexts [38] showed that implicit biases related to gender, race, and weight, have significant implications for clinical judgment and medical behaviour, influencing both the quality of care provided and the clinical decisions made (e.g., physicians being less certain of the diagnosis of coronary heart disease for middle-aged women, who were thus twice as likely to receive a mental health diagnosis than their male counterparts). Although most of the studies reviewed focused on the effects of ethnicity, the findings also highlighted that gender has an impact on clinical assessment, diagnostic delays and variation in treatment quality.

Gender stereotypes are also present in occupational and academic settings, where they are associated with biases in evaluations of performance, leadership ability, professional commitment and dedication, disadvantaging women and gender non-conforming individuals [28]. Once again, the hospital environment provides numerous examples of gender discrimination. Particularly about nurses, several stereotypes exist, starting from the idea that male nurses are merely “doctor wannabes” and undermining their professional choice. Assumptions include that, as men, they are less capable of providing care and, moreover, that their gender and profession imply they are homosexual or sexual predators. The latter constitutes the most widespread and harmful stereotype [39]. The underlying idea is that women are more suited to “caring,” while men are associated with “healing.” This stereotype is present not only in the perceptions of patients but also among healthcare staff themselves. Women tend to perceive their skills as “unqualified” and are expected to be accommodating and supportive, placing others’ needs before their own. Men, on the other hand, are expected to demonstrate greater strength and are therefore assigned to manage violent or complex patients. Moreover, men often emphasize their masculine traits to distance themselves from the feminine stereotype and to assert hegemonic masculinity, whereas women tend to downplay the discrimination they experience, framing it as systemic and therefore inevitable [21].

Several reviews highlighted that these mechanisms operate across contexts and are reinforced by cultural norms, rendering them resistant to individual awareness and correction. Importantly, these cognitive processes were not conceptualized as discrimination per se, but as antecedent mechanisms that increase the likelihood of differential treatment when translated into behaviour and institutional practices.

#### 3.1.2. Structural and Overt Discrimination

##### Societal Gender-Discrimination

The included reviews identified systematic discrimination manifested through institutional barriers, governmental policies, and organizational practices that create measurable disparities in opportunities, outcomes, and treatment based on gender. This category encompasses formalized discriminatory mechanisms documented across multiple professional and societal contexts, including pay disparities, advancement barriers, and policy-based exclusions. Structural elements appear to be components within broader discrimination frameworks, indicating the interconnected nature of discrimination categories across the evidence base. Evidence from son-preference societies demonstrates the most extreme manifestations of structural gender discrimination. Pennington and colleagues [27] documented the phenomenon of “missing women” within son-preference countries, revealing how societal structures perpetuate systematic gender-based mortality through mechanisms including sex-selective abortion and differential care provision. This analysis quantified women who would be alive absent gender preferences within specific national contexts, illustrating how deeply embedded cultural values translate into measurable demographic consequences. Similarly, Ali et al. [31] documented comprehensive societal exclusion mechanisms where gender discrimination becomes institutionalized through the absence of protective policies and maintenance of discriminatory status quo conditions (such as not being allowed to participate in elections). These structural barriers systematically exclude women from decision-making positions and professional environments while confining them to traditionally assigned domestic roles, demonstrating how societal-level discrimination operates through both active exclusion and passive policy neglect.

##### Workplace Structural Discrimination

Once again, the healthcare system presents itself as a place where this type of discrimination can be clearly seen. Systematic discrimination within healthcare systems particularly affects transgender populations through deliberate underinvestment in specialized services. Chong et al. [23] documented how structural deficiencies in transgender-specific healthcare create disproportionate barriers for vulnerable subgroups, including sex workers, and compel treatment abandonment among youth populations seeking HIV care through restrictive legislation and complex insurance policies that create systematically insurmountable obstacles to essential healthcare access. The evidence demonstrates how healthcare discrimination operates through multiple mechanisms: inadequate service provision, regulatory barriers, and financial exclusion that collectively deny access to gender-affirming care and create conditions necessitating dangerous workarounds.

Multiple reviews documented systematic discrimination within healthcare organizations, revealing complex patterns affecting both women and men depending on hierarchical positioning and professional roles. Gupta et al. [32] found that while women generally reported higher workplace discrimination rates, gender discrimination represented the most prevalent form of mistreatment for both men and women within gynaecological surgery settings, indicating that structural discrimination operates through professional context rather than gender alone. Among physicians, systematic discrimination disproportionately affects women through both hierarchical exclusion and differential work assignment practices, with Lyons et al. [29] documenting substantially higher discrimination rates among female physicians compared to male colleagues, manifesting through exclusion from surgical opportunities and assignment to traditionally gendered responsibilities such as administrative duties rather than clinical practice. These organizational patterns demonstrate how structural discrimination becomes operationalized through institutional hierarchies, role assignments, and differential opportunity provision that systematically disadvantage specific gender groups within professional contexts.

#### 3.1.3. Interpersonal and Subtle Discrimination

##### Microaggression as Discriminatory Behaviour

Often, structural and overt forms of discrimination stem from more subtle forms of discrimination, which occur at the interpersonal level or in the form of microaggressions. Microaggressions are a subtle and often ambiguous form of discrimination that can lead to various outcomes in terms of adaptation, particularly psychological adaptation. Although they are highly correlated with overt discrimination, the two forms appear to have complex relationships [34]. As shown in the review by Pennington and colleagues [27], mechanisms of selection and preference for male children are visible in everyday behaviours, such as providing less care to girls than to boys. This relative neglect also affects access to family resources, which are not limited to food or money but also include parental attention. The result is an increased risk of excessive mortality and reduced survival of women across the life course, from childhood to adulthood. These practices are closely connected to the way in which the birth of a boy is celebrated compared to that of a girl, and to the social status a woman gains depending on the sex of her child. The perpetuation of these dynamics, seen as “normal” and culturally accepted, makes this type of discrimination particularly difficult to identify and combat. Men can ignore their wives’ views and opinions, treating them as sexual objects with no rights or identity outside of the man who makes decisions for them, creating a dynamic in which women are unable to act without men, forced into submissive roles [31]. This aspect highlights the dual nature of subtle discrimination: not only can it be perceived (in the form of judgements, behaviours or attitudes) as directed towards a specific category, but it can also be internalised: individuals absorb society’s negative attitudes about their own group and perpetuate them [26].

##### Workplace Interpersonal Discrimination

These behaviours are not always linked to explicit beliefs but also serve as strategies to reinforce identity. In the case of eSports, for example, there are numerous examples of interpersonal discrimination against women. Female players experience interpersonal discrimination such as receiving more negative feedback or no feedback at all, when speaking with their own voices. In response, they adopt protective strategies such as using voice distortion, avoiding microphones, or choosing neutral nicknames. These practices serve both as forms of gatekeeping and as mechanisms to preserve male dominance in gaming. Despite presenting itself as meritocratic, the eSports world is structured in ways that advantage men, forcing women to negotiate their gender identity and, in doing so, sometimes reinforcing existing stereotypes [30].

Similar dynamics emerge in a professional context as well. In the workplace, women suffer forms of microaggression related to gender (and sometimes ethnicity); examples include being asked to “smile more” or “dress more femininely”, or, if we go back to hospitals, not being considered suitable for surgical roles. Subtle discrimination also arises in interactions with staff, where women often need to repeat requests several times, tend to “pre-apologise” for legitimate demands, and are interrupted more often than male colleagues. Moreover, women are frequently excluded from informal or social meetings where key decisions are made [29,32]. The case of healthcare settings is particularly important also from the patients’ perspective. Transgender people often feel deprived of their dignity due to repeated pressures to come out, or to comply with medical resistance against gender affirmation and fertility choices, even when they clearly express a different wish. Several studies document instances in which clinicians asked irrelevant questions about patients’ gender or conducted unnecessary genital examinations, making them feel violated. Misuse of pronouns represents another significant form of discrimination, perceived as disrespectful and as a refusal to affirm their gender identity, especially when accompanied by dismissive comments such as “it’s just a phase” [23].

##### Sexuality-Based Gender Discrimination

Sexuality is another sphere where subtle discrimination emerges in particularly harmful ways, especially for women. Victims are frequently marginalised, excluded from social groups, or openly judged for their physical appearance and clothing. This form of behaviour is the outcome of a sexual double standard that values relationships differently depending on gender, and which functions as a form of control. Fearing stigmatisation, many women suppress their own desires, even when judgements are based solely on perceptions rather than facts [35].

Ultimately, the cumulative effect of such dynamics has a profound impact on the mental health of marginalised groups. Heterosexism, directed against LGBTQIA+ individuals, has emerged as one of the most concealed yet socially legitimised forms of discrimination. Sexism, in turn, shows negative effects of similar magnitude to racism. The meta-analysis conducted by Emmer and colleagues [33] highlights the pervasiveness of these mechanisms: subtle discrimination is frequent, chronic, and, according to their analysis, exerts the strongest impact on mental health (see Theme 2).

### 3.2. Theme 2: Gender-Based Discrimination Outcomes

This theme synthetizes evidence on consequences of gender-based discrimination on health (both mental and physical) and professional and social functioning. Across reviews, the outcomes emerged as cumulative and multidimensional, shaped by the interaction between individual vulnerability, interpersonal exposure and structural constraints.

#### 3.2.1. Mental Health Consequences

##### Prevalence and Core Mental Health Outcomes

Among all the negative effects associated with gender discrimination, deterioration of mental health has emerged as the most common and frequent outcome. Poorer mental health is identified as the most frequent consequence of gender discrimination, with several studies specifically measuring anxiety and depression through validated scales [40]. Beyond these conditions, gender discrimination was also linked to a wider range of detrimental outcomes, such as psychological distress, occupational stress, feelings of vulnerability, discomfort, fatigue, rage, alienation, and reduced self-concept. Importantly, the association between discrimination and psychological distress extended beyond gender, as all studies examining other forms of discrimination (e.g., based on sexual identity) also reported similar effects.

##### Contextual and Relational Mechanisms

Contextual factors such as limited decision-making power and reduced autonomy, particularly for women in family settings, were found to exacerbate disadvantage and contribute to negative mental health outcomes. Mental health plays a central role both as a mechanism and as a correlate in understanding the effects of stigma [26]. Internalized stigma emerges as a stronger predictor of psychological distress than perceived discrimination, reinforcing the idea that mental health mediates the impact of stigma on individual and relational well-being. Within this framework, poorer relationship functioning is understood because of stigma, largely mediated by processes such as emotion dysregulation, heightened negative affect, and psychological distress. Stressors like stigma are filtered through cognitive appraisal processes but ultimately shape emotional responses that spill over into intimate relationships, consistent with the “social stress hypothesis”. Discrimination directly undermines mental health, as is highlighted in experimentally manipulated discrimination analysed by Emmer and colleagues [33], showing a negative overall effect, even after accounting for study quality, region, and participant characteristics. Importantly, the effect was not uniform across mental health domains. Discrimination most strongly increased externalizing outcomes such as anger and hostility, followed by distress-related outcomes such as anxiety and negative affect. In contrast, no significant immediate effects were observed for well-being indicators (e.g., life satisfaction, positive affect) or self-directed outcomes (e.g., self-esteem, shame). The strength of these effects was moderated by the type and context of discrimination: pervasive, systemic discrimination exerted a larger negative impact than single incidents, and marginalized groups (e.g., women in sexism studies) experienced stronger effects compared to non-marginalized groups. Across types of discrimination, heterosexism yielded the most pronounced effect, followed by racism and sexism, while ageism, body-related, and status-related discrimination did not reach statistical significance.

##### Transgender and Gender Non-Conforming Populations

The link between gender discrimination and mental health is particularly important when considering the transgender and gender non-conforming population. As Chong and colleagues [23] report in their systematic review, transgender youths’ encounters with healthcare systems have profound mental health consequences, reflected across six interrelated themes. Medical settings often exacerbate gender dysphoria, particularly when procedures force attention to incongruent anatomical features, leaving participants distressed and destabilized. The hostile nature of healthcare access was also linked to heightened anxiety, depression, and emotional burden, with fears of discrimination, ineffective treatment, and clinician ignorance compounding distress. In extreme cases, these pressures contributed to suicidality and self-harm, with some youths describing fertility preservation or interruptions in hormone therapy as triggers for harmful behaviours. Experiences of stigma, objectification, and intrusive questioning within medical contexts further produced trauma and feelings of dehumanization, reinforcing mistrust and avoidance of care. This manifested in help-seeking inertia, where youths censored or concealed needs for fear of rejection, withdrawal of care, or being judged “imperfect,” leaving them reluctant to seek even essential support. Yet, the review also highlights resilience and post-traumatic growth: affirming encounters with competent, respectful clinicians, solidarity within the transgender community, and supportive relationships provided crucial buffers, fostering trust, resilience, and commitment to health. Similar results were found in Lin et al. [25]. In their review a strikingly high prevalence of mental health disorders among transgender and gender non-conforming individuals in China is reported, with depression (32–54.5%) and anxiety (28.5–51%) consistently reported at rates far exceeding the general population. Substance use was frequently comorbid with these conditions, particularly among transgender women sex workers, where sexualized drug use was strongly linked to depressive and anxious symptomatology. Alarmingly, suicidality and self-harm emerged as pervasive concerns: suicidal ideation affected up to half of participants, while nearly one in five reported suicide attempts, with transgender women exhibiting higher risk than transgender men. Stress-related disorders were also prominent, with almost half of transgender women experiencing moderate-to-severe psychological distress and one quarter meeting criteria for Post-Traumatic Stress Disorder. These outcomes were explained through the minority stress model, which situates discrimination, violence, familial rejection, barriers to gender-affirming care, and hostile structural conditions as key drivers of poor mental health. Structural vulnerability was compounded by economic hardship and reliance on sex work, which increased exposure to violence and substance use. Despite this substantial burden, mental health service utilization remained critically low, with fewer than one in four transgender women accessing care in the past year, primarily due to fears of stigma, discrimination, and breaches of confidentiality. Yet, the review also highlights resilience and protective factors: social support networks within families and transgender communities, individual resilience, and access to gender-affirming hormone therapy were all associated with reduced symptoms and improved wellbeing. Collectively, these findings underscore the severe mental health disparities faced by transgender and gender non-conforming individuals in China, while also pointing to the critical role of affirming care and social support in fostering resilience.

#### 3.2.2. Physical Health Implications

##### Life-Course and Demographic Implications

Gender discrimination manifests in profound demographic consequences across the life course. Structural neglect of girls and women, through reduced access to nutrition, healthcare, education, and economic resources, translates into excess female mortality from birth through old age. This neglect is compounded by skewed sex ratios at birth, driven by prenatal sex selection in son-preference societies. Whereas the natural ratio is 105–107 males per 100 females, countries such as China (up to 121.2), India (113.1), Armenia (126), and Azerbaijan (119) have recorded significantly inflated figures. Gender inequality undermines women’s health, spanning nutrition, healthcare access, reproductive rights, and exposure to violence. Women frequently suffer neglect in health and nutrition, lacking autonomy over reproductive decisions such as birth spacing and access to adequate prenatal and postnatal care, factors that contribute directly to elevated maternal mortality and morbidity [27,31]. Nutritional discrimination within households further exacerbates vulnerability, as men and boys are often prioritized in food distribution, leaving women and girls at higher risk of malnutrition. Son preference intensifies these inequalities, creating pressure to produce male children, fuelling illegal sex-selective abortions, and reinforcing neglect of daughters’ health, while women who bear sons are often granted greater acceptance and status.

##### Reproductive, Maternal and Child Health

Gender-based violence compounds the problem, with widespread physical, sexual, and emotional abuse producing both direct physical harms (injuries, chronic pain, reproductive health complications) and indirect consequences through psychological trauma. Structural barriers to healthcare remain pervasive: women’s mobility is restricted by cultural norms requiring male permission, and the shortage of female providers further limits access. Maternal health disparities reflect these inequalities, with high mortality linked to inadequate skilled attendance at birth, insufficient antenatal and postnatal care, early and forced marriages, and frequent, poorly spaced pregnancies due to lack of contraception. Maternal autonomy has profound consequences for both child and maternal health. Reduced decision-making power among women is consistently associated with higher rates of infant and child mortality, including neonatal, post-neonatal, and childhood deaths. Children of women with low autonomy are more likely to experience malnutrition, reflected in stunting, wasting, and underweight, as well as greater vulnerability to acute conditions such as diarrhoea and respiratory infections. Gender-based nutritional disparities are particularly pronounced: girls in low-autonomy households are disproportionately malnourished compared to boys. Maternal health outcomes are similarly compromised, with limited autonomy linked to chronic energy deficiency, low BMI, anaemia, and adverse birth outcomes such as low birth weight. While not always directly measured, restricted autonomy also heightens maternal mortality risk by reducing access to appropriate healthcare during pregnancy and childbirth [42].

##### Physiological Stress and Chronic Diseases

Moreover, discrimination seems to be consistently linked to adverse cardiovascular outcomes [41]. Evidence shows significant associations with elevated blood pressure, reduced heart rate variability, heightened stress biomarkers (e.g., cortisol, CRP, interleukins), and direct disease indicators such as coronary artery calcification, endothelial dysfunction, and increased risk of cardiovascular events. Experimental and longitudinal studies further support a causal link, showing that discriminatory stressors trigger acute physiological responses and predict long-term disease risk. Similar patterns, though less studied, were found for weight and sexual orientation discrimination, which were associated with inflammatory markers, obesity progression, and poor glycaemic control.

##### Transgender-Specific Physical Health Risks

Transgender youths face significant physical health risks, not because of their identities, but due to systemic barriers and discrimination within healthcare [23]. Limited access to safe, affirming care often leads to high-risk practices such as self-administering unregulated hormones, industrial silicone injections, or needle sharing, with severe risks of infection and overdose. Fear of stigma further drives avoidance of essential preventive care, including HIV testing and reproductive health screenings, resulting in delayed diagnoses and advanced disease. Even when accessing gender-affirming care, youths report distress from side effects, fertility preservation procedures, and anxiety about surgical outcomes, while comorbid conditions such as HIV and STIs are exacerbated by stigma and lack of education. Negative clinical encounters, including unnecessary, painful, or invasive procedures, multiply these harms, producing preventable infections, unmanaged side effects, and overall poorer health outcomes.

#### 3.2.3. Professional and Social Functioning

##### Structural Constraints on Professional Advancement

The systematic reviews synthesized in this umbrella review document profound impairments to professional and social functioning arising from gender-based discrimination across diverse occupational domains and sociocultural contexts. These impairments operate through interconnected mechanisms at individual, organizational, and societal levels, systematically constraining professional advancement, economic participation, and social autonomy. In academic medicine, women physicians experience substantially delayed progression to senior ranks, requiring 19 years to achieve associate or full professor status compared to 16 years for male counterparts, while 44% of women never attain leadership positions versus 30% of men [29]. Similar patterns emerge in academic surgery and corporate sectors, where systemic bias in recruitment and promotion manifests through predominantly male leadership selecting demographically similar candidates while applying elevated standards to female applicants [28,43]. These structural barriers are compounded by exclusion from informal, male-dominated social networks that provide essential access to promotion opportunities and career advancement support [28].

##### Differential Treatment and Harassment

Conversely, male nurses experience a “glass escalator” effect with faster promotion and more opportunities based on gender rather than merit, including instances of less experienced males being offered advanced roles over more qualified female colleagues [21]. In resource-constrained contexts, women face fundamental restrictions including limited educational attainment, mobility constraints, and sex-segregated occupational choices [31], while female entrepreneurship remains severely restricted due to capital unavailability and restrictive cultural customs. In societies characterized by profound gender discrimination, women’s professional functioning is constrained by severe autonomy restrictions, including lack of freedom to move outside the home unaccompanied and prohibition from working without spousal permission [42]. Economic disparities constitute a fundamental dimension of impaired professional functioning, with women physicians experiencing persistent adjusted salary gaps from career onset [29] and employed women across sectors facing systematically lower wages [31]. The professional environment is characterized by pervasive harassment, with sexual harassment affecting 27.6% to 70.9% of clinicians in obstetrics and gynaecology, workplace discrimination experienced by 57.0% to 67.2% of women, and microaggressions reported by 83.2% of respondents; fear of retaliation prevents 33.5% to 40.2% of harassment victims from reporting incidents [32]. Across medical specialties, 65% of women versus 10% of men experience gender discrimination and 30% versus 6% experience sexual harassment, correlating with diminished career satisfaction and adverse mental health outcomes including elevated rates of depression and suicide attempts [29]. Female academic surgeons experience heightened burnout attributable to gender bias and work-home conflicts [43], with particularly pronounced effects for women from underrepresented communities facing intersectional discrimination. In nursing, gender dynamics manifest distinctly with male nurses facing exclusion from specialties like Paediatrics and Midwifery, mistreatment including disproportionate assignment of physically demanding tasks, and social hostility with deliberate exclusion from workplace social activities [39]. Male nurses also face challenging social perceptions where nursing is viewed as having lower social status, affecting personal relationships [21,39]. However, male nurses simultaneously receive “unearned respect” and privileged positioning, are treated as a “protected minority” receiving less blame for mistakes, and benefit from patriarchal culture prioritizing their success; female nurses experience career interruptions due to gendered caring responsibilities, with skills devalued after having children and decreased access to training opportunities [21]. Healthcare professionals’ implicit biases significantly compromise professional functioning through distorted clinical judgment, with physicians and nurses exhibiting biases across patient characteristics [38]: patient characteristics influence diagnostic certainty and treatment recommendations, including reduced certainty in coronary heart disease diagnoses for middle-aged women and decreased likelihood of prescribing appropriate medications for women presenting identical symptoms.

##### Legal Reinforcement and Social Autonomy

Gender stereotypes fundamentally distort professional evaluation, with descriptive stereotypes positing that women lack agentic traits necessary for leadership creating perceived “lack of fit” and generating bias in promotion decisions [28], while prescriptive stereotypes result in successful women facing social penalties, producing a “double bind” wherein women are perceived as either professionally incompetent or socially penalized for demonstrating competence. Legal jurisprudence reflects these dynamics, with landmark cases demonstrating penalties for assertive behaviour and discrimination based on motherhood status [36], while meta-analytic evidence indicates that despite equivalent leadership effectiveness, women receive less favourable evaluations, particularly in masculine-stereotyped domains. Organizational cultures characterized by masculinity compromise professional functioning through work structures equating commitment with extensive hours and uninterrupted career trajectories [28], while social norms imposing disproportionate domestic responsibilities on women, who dedicate 31 h weekly to household tasks and childcare versus 19 h for men, compel women to select career tracks prioritizing work-life balance and to perceive children as career barriers [29]. In contexts characterized by severe gender discrimination, women’s social functioning is profoundly constrained by restrictions on autonomy, with low societal status manifesting through objectification and denial of independent decision-making authority [31]. Constraints on freedom of movement, including purdah practices (i.e., social and cultural norms that regulate gender segregation, particularly by restricting women’s visibility, mobility, and interactions in public spaces), severely limit capacity to engage in social activities, access healthcare, and participate in economic life [42]. Synthesis of evidence establishes that women’s diminished autonomy in social and domestic spheres significantly associates with poorer mental and physical health for women and elevated morbidity and mortality for their children, even after adjusting for socioeconomic status [42]. Legal and institutional frameworks frequently reinforce rather than remediate gender-based barriers, with analysis revealing that nearly half of significant court decisions upheld gender stereotypes, thereby perpetuating traditional roles and hindering professional advancement [36].

## 4. Discussion

The synthesis of the papers analysed in this umbrella review highlights that gender discrimination is a multidimensional phenomenon, which is rooted in institutional, interpersonal, and cognitive processes. Discrimination manifests itself as a systemic configuration of barriers linked to social and cultural structures and norms, rather than an isolated occurrence; according to the reviewed papers, gender-based discrimination manifests itself through institutional barriers, governmental policies, and organizational practices creating measurable disparities in opportunities, outcomes, and treatments. In this structural context, implicit biases become discriminatory practices; for this reason, it is necessary to consider structural discrimination as the main object of analysis, recognising its key role in creating the conditions that enable and fuel other manifestations of gender discrimination in various areas. Yet, there is still considerable theoretical fragmentation in the definition and functioning of gender discrimination, as highlighted by the analysis conducted. Critical divergence emerged regarding conceptualization and measurement protocols. De la Torre-Pérez and colleagues [40] addressed this heterogeneity by proposing a dual-component construct encompassing “undervaluation” and “different treatment” following systematic analysis of definitional inconsistencies; all of this significantly hinders comparability between studies, meta-analytic aggregation, and theoretical integration. The absence of a common framework limits the possibility of research capturing the full spectrum of discrimination, from overt institutional exclusion to the internalisation of social hierarchies at the cognitive level. Although most reviews have focused on discrimination against women, we know that gender discrimination operates in multiple directions, including against LGBTQIA+ groups and men. Through gender discrimination, the application of binary hierarchy norms is perpetuated, in line with contemporary perspectives on intersectionality, according to which discrimination is also shaped through overlapping systems of gender, sexual orientation, class, ethnicity, disability and cultural norms.

Notably, the evidence synthesized in the included reviews predominantly focused on the detrimental effects of gender discrimination on marginalized groups, while comparatively little attention was devoted to examining potential advantages accruing to more privileged groups, highlighting a systematic gap in the existing secondary literature.

Despite empirical evidence, several methodological and geographical limitations inhibit this field of research and, consequently, the present umbrella review. Most of the primary studies in the reviews analysed are based on cross-sectional designs [25,34], which is why causal inferences or longitudinal assessments of the consequences of discrimination cannot be made. Experimental paradigms, such as those synthesised by Emmer and colleagues [33], are also not yet widely applied and rarely include long-term follow-up. It should also be noted that current scientific evidence is heavily biased towards Western research contexts, particularly in the United States [41,43]. This limited representation of both non-Western countries and genders other than the binary division requires greater cultural contextualisation and demographic inclusivity in future research.

From a methodological point of view, research is dominated by self-assessment measures and convenience samples, particularly among university students; this means there is a risk of reproducing sampling bias, limited ecological validity and reduced generalisability of results in different contexts. It is necessary to begin applying a methodological pluralism that integrates implicit measures, behavioural paradigms, and qualitative projects that allow context-dependent differences to be captured. Finally, this umbrella review revealed a lack of systematic research on interventions. Numerous studies have documented the prevalence and psychological consequences, but few have systematically evaluated the effectiveness of strategies aimed at reducing prejudice and mitigating discrimination. The available evidence [31] supports the value of multilevel interventions; however, rigorous evaluation of their long-term effectiveness remains limited. The absence of sustained intervention research highlights a structural gap between descriptive evidence and transformative practice: bridging this gap requires coordinated efforts across disciplines so that research data can be transformed into institutional governance policies that enable structural change. The limited availability of effective intervention evidence should also be interpreted considering the level at which gender discrimination operates. Much of the discrimination documented in the included reviews occurs at institutional and societal levels, where individual- or group-focused interventions are inherently more difficult to design, implement, and evaluate.

## 5. Conclusions

This umbrella review aims to provide a comprehensive summary of the scientific evidence on gender discrimination, highlighting the interconnected mechanisms through which it operates. Discrimination is not only a social inequality, but also a psychological and systemic process that perpetuates inequality through normalisation, internalisation and institutional reproduction. The findings underscore the need for an integrated approach that links theoretical refinement, methodological innovation, and policy translation. Future research should prioritise cross-cultural and longitudinal projects, standardise measurement frameworks, and evaluate the effectiveness of intervention programmes. Broadening the view and perspective, addressing gender discrimination requires going beyond individual-level change to systemic transformation, in which institutions, cultural narratives, and cognitive schemas are simultaneously restructured to promote equity. It is therefore necessary to emphasise the role of psychology in highlighting how structural inequality is embodied in cognition and health. Understanding these mechanisms is a prerequisite for designing interventions that not only reduce discrimination but also promote sustainable social and psychological well-being.

## Figures and Tables

**Table 1:** Thematic Categories.

Umbrella Categories	Dimensions	Papers
Discrimination manifestation	Structural and overt discrimination	[23,27,29,31,32]
	Interpersonal and subtle discrimination	[23,26,27,29,30,31,32,33,34,35]
	Implicit biases and stereotyping mechanisms	[21,24,27,28,31,36,37,38,39]
Health and professional outcomes	Mental health consequences	[23,25,26,33,40]
	Physical health implications	[23,27,31,41,42]
	Professional and social functioning	[21,26,28,29,31,32,36,38,39,42,43]

## Data Availability

No new data were created or analyzed in this study. Data sharing is not applicable.

## Supplementary Materials

The following supporting information can be downloaded at: https://www.mdpi.com/article/10.3390/ijerph23010103/s1, File S1: UnveilGBD Umbrella Review Data Table.

## Citation Diversity Statement

Following the work from Zurn and colleagues [44], to proactively consider choosing references that reflect the diversity of the field in thought, form of contribution, gender, race, ethnicity, and other factors, I used databases that store the probability of a first name being carried by a woman. As per gender diversity, by this measure and excluding self-citations to the first and last authors of our current paper, this paper contains 33.3% woman(first)/woman(last), 18.0% man/woman, 30.0% woman/man, and 18.0% man/man. This method is limited in that (a) names, pronouns, and social media profiles used to construct the databases may not, in every case, be indicative of gender identity and (b) it cannot account for intersex, non-binary, or transgender people.

## References

[1] Obodo C.A., Leal Filho W., Marisa Azul A., Brandli L., Lange Salvia A., Gökçin Özuyar P., Wall T. (2021). Gender-Related Discrimination. Reduced Inequalities.

[2] Lausi G., Cricenti C., Mari E., Burrai J., Quaglieri A., Giannini A.M., Barchielli B. (2024). An explorative study on consequences of abuse on psychological wellbeing and cognitive outcomes in victims of gender-based violence. Front. Psychol..

[3] Greenwald A.G., Banaji M.R. (1995). Implicit social cognition: Attitudes, self-esteem, and stereotypes. Psychol. Rev..

[4] SteelFisher G.K., Findling M.G., Bleich S.N., Casey L.S., Blendon R.J., Benson J.M., Sayde J.M., Miller C. (2019). Gender discrimination in the United States: Experiences of women. Health Serv. Res..

[5] Sue D.W., Capodilupo C.M., Torino G.C., Bucceri J.M., Holder A.M.B., Nadal K.L., Esquilin M. (2007). Racial microaggressions in everyday life: Implications for clinical practice. Am. Psychol..

[6] Cordellieri P., Paoli E., Giannini A.M., Lausi G. (2024). From verbal to physical violence: The different severity perception of stalking behaviors. Curr. Psychol..

[7] Heilman M.E., Caleo S., Manzi F. (2024). Women at Work: Pathways from Gender Stereotypes to Gender Bias and Discrimination. Annu. Rev. Organ. Psychol. Organ. Behav..

[8] Colella A. (2018). The Oxford Handbook of Workplace Discrimination.

[9] De Vries A.L.C., Kreukels B.P.C., Steensma T.D., McGuire J.K., Kreukels B.P.C., Steensma T.D., De Vries A.L.C. (2014). Gender Identity Development: A Biopsychosocial Perspective. Gender Dysphoria and Disorders of Sex Development.

[10] Eckes T., Trautner H.M. (2012). The Developmental Social Psychology of Gender.

[11] Egan S.K., Perry D.G. (2001). Gender identity: A multidimensional analysis with implications for psychosocial adjustment. Dev. Psychol..

[12] Fibbi R., Midtbøen A.H., Simon P. (2021). Concepts of Discrimination. Migration and Discrimination.

[13] Hudson S.T.J., Myer A., Berney E.C. (2024). Stereotyping, prejudice, and discrimination at the intersection of race and gender: An intersectional theory primer. Soc. Personal. Psychol. Compass.

[14] Junça-Silva A., Ferreira N. (2025). Workplace micro-aggressions and affective consequences: The moderating role of emotional contagion. Curr. Psychol..

[15] McAllister A., Fritzell S., Almroth M., Harber-Aschan L., Larsson S., Burström B. (2018). How do macro-level structural determinants affect inequalities in mental health?—A systematic review of the literature. Int. J. Equity Health.

[16] Domínguez S., Embrick D.G. (2020). Racial microaggressions: Bridging psychology and sociology and future research considerations. Sociol. Compass.

[17] Hatzenbuehler M.L., Nolen-Hoeksema S., Dovidio J. (2009). How Does Stigma “Get Under the Skin”?: The Mediating Role of Emotion Regulation. Psychol. Sci..

[18] Mezzina R., Gopikumar V., Jenkins J., Saraceno B., Sashidharan S.P. (2022). Social Vulnerability and Mental Health Inequalities in the “Syndemic”: Call for Action. Front. Psychiatry.

[19] Schurz M., Radua J., Tholen M.G., Maliske L., Margulies D.S., Mars R.B., Sallet J., Kanske P. (2021). Toward a hierarchical model of social cognition: A neuroimaging meta-analysis and integrative review of empathy and theory of mind. Psychol. Bull..

[20] Gawronski B., Ledgerwood A., Eastwick P.W. (2022). Implicit Bias ≠ Bias on Implicit Measures. Psychol. Inq..

[21] Gauci P., Luck L., O’Reilly K., Peters K. (2023). Workplace gender discrimination in the nursing workforce—An integrative review. J. Clin. Nurs..

[22] Acosta J., Chinman M., Tharp A., Baker J., Flaspohler P., Fortson B., Kerr A., Lamont A., Meyer A., Smucker S. (2022). Development and pilot test of criteria defining best practices for organizational sexual assault prevention. Prev. Med. Rep..

[23] Chong L.S.H., Kerklaan J., Clarke S., Kohn M., Baumgart A., Guha C., Tunnicliffe D.J., Hanson C.S., Craig J.C., Tong A. (2021). Experiences and Perspectives of Transgender Youths in Accessing Health Care: A Systematic Review. JAMA Pediatr..

[24] Campbell M., Hinton J.D.X., Anderson J.R. (2019). A systematic review of the relationship between religion and attitudes toward transgender and gender-variant people. Int. J. Transgenderism.

[25] Lin Y., Xie H., Huang Z., Zhang Q., Wilson A., Hou J., Zhao X., Wang Y., Pan B., Liu Y. (2021). The mental health of transgender and gender non-conforming people in China: A systematic review. Lancet Public Health.

[26] Doyle D.M., Molix L. (2015). Social Stigma and Sexual Minorities’ Romantic Relationship Functioning: A Meta-Analytic Review. Pers. Soc. Psychol. Bull..

[27] Pennington A., Maudsley G., Whitehead M. (2023). The impacts of profound gender discrimination on the survival of girls and women in son-preference countries—A systematic review. Health Place.

[28] Van’t Foort-Diepeveen R.A., Argyrou A., Lambooy T. (2021). Holistic and integrative review into the barriers to women’s advancement to the corporate top in Europe. Gend. Manag..

[29] Lyons N.B., Bernardi K., Olavarria O.A., Shah P., Dhanani N., Loor M., Holihan J.L., Liang M.K. (2021). Gender Disparity Among American Medicine and Surgery Physicians: A Systematic Review. Am. J. Med. Sci..

[30] Rogstad E.T. (2022). Gender in eSports research: A literature review. Eur. J. Sport Soc..

[31] Ali T.S., Ali S.S., Nadeem S., Memon Z., Soofi S., Madhani F., Karim Y., Mohammad S., Bhutta Z.A. (2022). Perpetuation of gender discrimination in Pakistani society: Results from a scoping review and qualitative study conducted in three provinces of Pakistan. BMC Women’s Health.

[32] Gupta A., Thompson J.C., Ringel N.E., Kim-Fine S., Ferguson L.A., Blank S.V., Iglesia C.B., Balk E.M., Secord A.A., Hines J.F. (2024). Sexual Harassment, Abuse, and Discrimination in Obstetrics and Gynecology: A Systematic Review. JAMA Netw. Open.

[33] Emmer C., Dorn J., Mata J. (2024). The immediate effect of discrimination on mental health: A meta-analytic review of the causal evidence. Psychol. Bull..

[34] Lui P.P., Quezada L. (2019). Associations between microaggression and adjustment outcomes: A meta-analytic and narrative review. Psychol. Bull..

[35] Miano P., Urone C. (2024). What the hell are you doing? A PRISMA systematic review of psychosocial precursors of slut-shaming in adolescents and young adults. Psychol. Sex..

[36] Castaño A., Fontanil Y., García-Izquierdo A. (2019). “Why Can’t I Become a Manager?”—A Systematic Review of Gender Stereotypes and Organizational Discrimination. Int. J. Environ. Res. Public Health.

[37] Degner J., Dalege J. (2013). The apple does not fall far from the tree, or does it? A meta-analysis of parent–child similarity in intergroup attitudes. Psychol. Bull..

[38] FitzGerald C., Hurst S. (2017). Implicit bias in healthcare professionals: A systematic review. BMC Med. Ethics.

[39] Ng M., See C., Ignacio J. (2024). Qualitative systematic review: The lived experiences of males in the nursing profession on gender discrimination encounters. Int. Nurs. Rev..

[40] De La Torre-Pérez L., Oliver-Parra A., Torres X., Bertran M.J. (2022). How do we measure gender discrimination? Proposing a construct of gender discrimination through a systematic scoping review. Int. J. Equity Health.

[41] Panza G.A., Puhl R.M., Taylor B.A., Zaleski A.L., Livingston J., Pescatello L.S. (2019). Links between discrimination and cardiovascular health among socially stigmatized groups: A systematic review. PLoS ONE.

[42] Pennington A., Orton L., Nayak S., Ring A., Petticrew M., Sowden A., White M., Whitehead M. (2018). The health impacts of women’s low control in their living environment: A theory-based systematic review of observational studies in societies with profound gender discrimination. Health Place.

[43] Ferrari L., Mari V., Parini S., Capelli G., Tacconi G., Chessa A., De Santi G., Verdi D., Frigerio I., Scarpa M. (2022). Discrimination Toward Women in Surgery: A Systematic Scoping Review. Ann. Surg..

[44] Zurn P., Bassett D.S., Rust N.C. (2020). The Citation Diversity Statement: A Practice of Transparency, A Way of Life. Trends Cogn. Sci..

